# Supplementary figures and images for: LAT1-Targeted Alpha Therapy Using 211At-AAMT for Bone and Soft Tissue Sarcomas
Source: Int J Mol Sci. 2025 Sep 4;26(17):8599. doi: 10.3390/ijms26178599 (PMC12428879; doi:10.3390/ijms26178599)

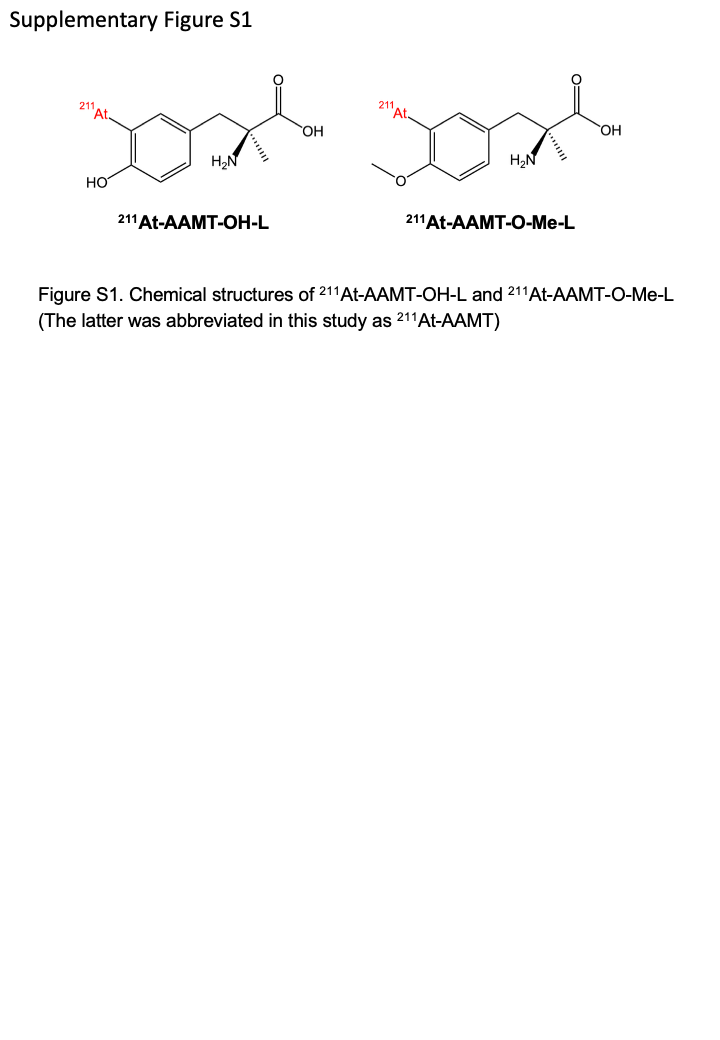

Supplement: Supplementary file 1 [file ijms-26-08599-s001.zip › Supplementary Figure S1.tiff]

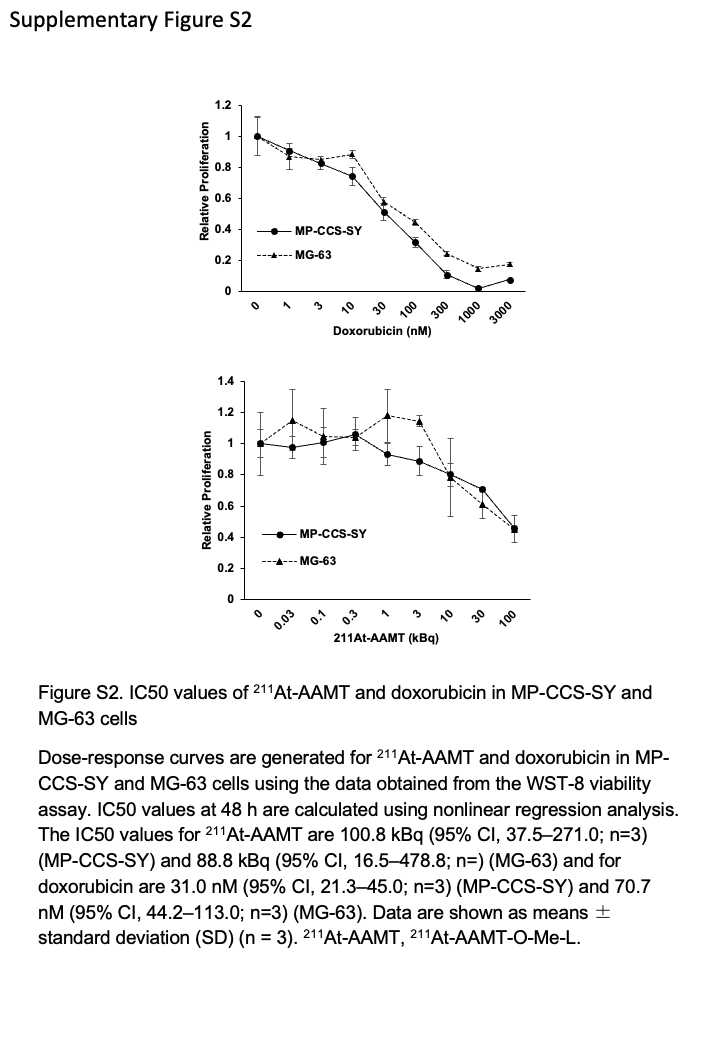

Supplement: Supplementary file 1 [file ijms-26-08599-s001.zip › Supplementary Figure S2.tiff]

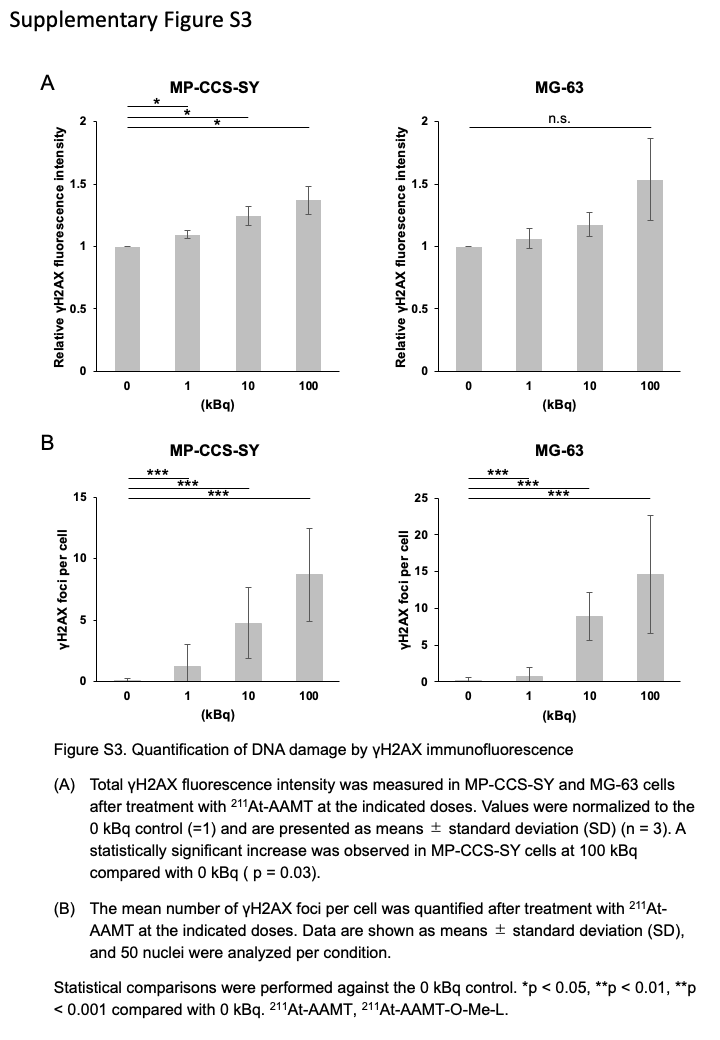

Supplement: Supplementary file 1 [file ijms-26-08599-s001.zip › Supplementary Figure S3.tiff]

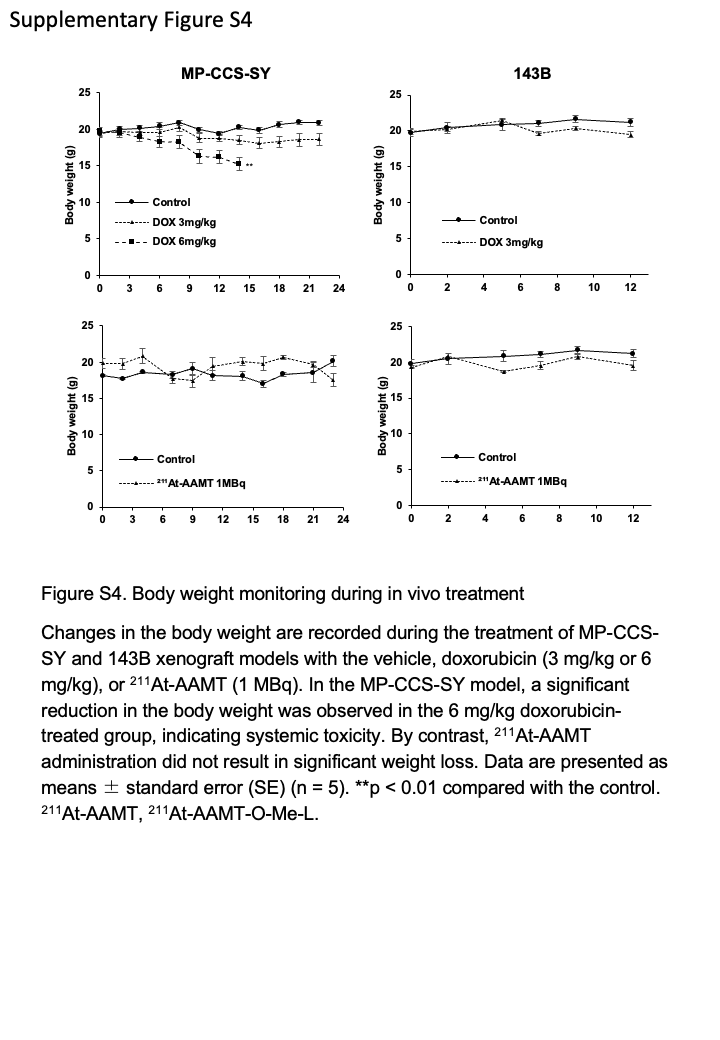

Supplement: Supplementary file 1 [file ijms-26-08599-s001.zip › Supplementary Figure S4.tiff]

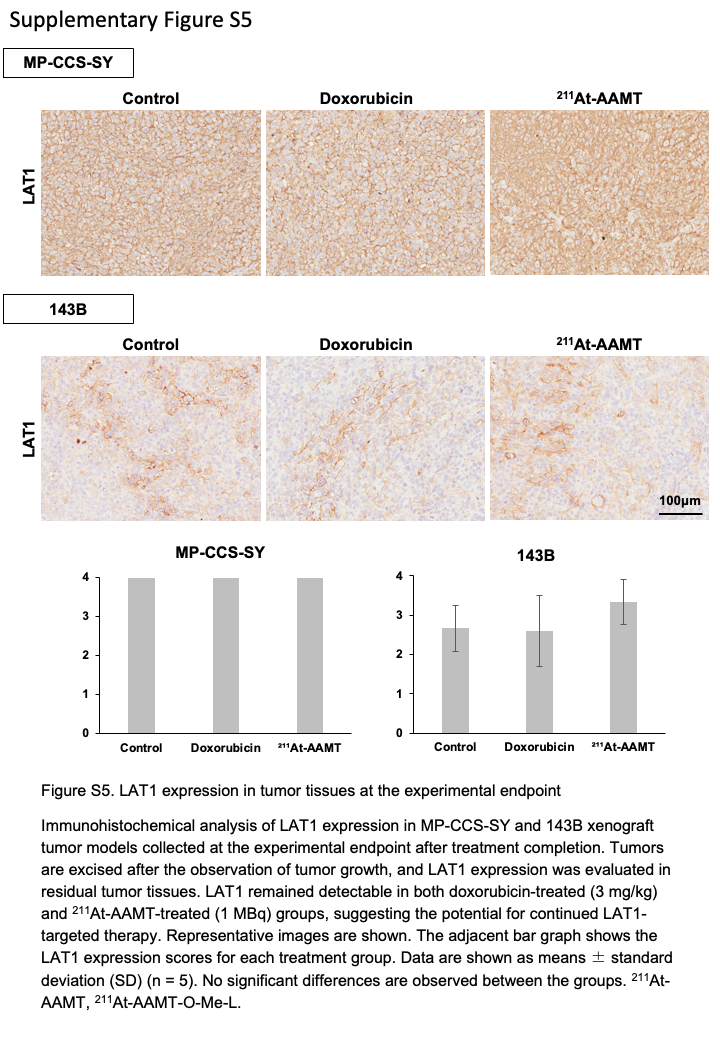

Supplement: Supplementary file 1 [file ijms-26-08599-s001.zip › Supplementary Figure S5.tiff]

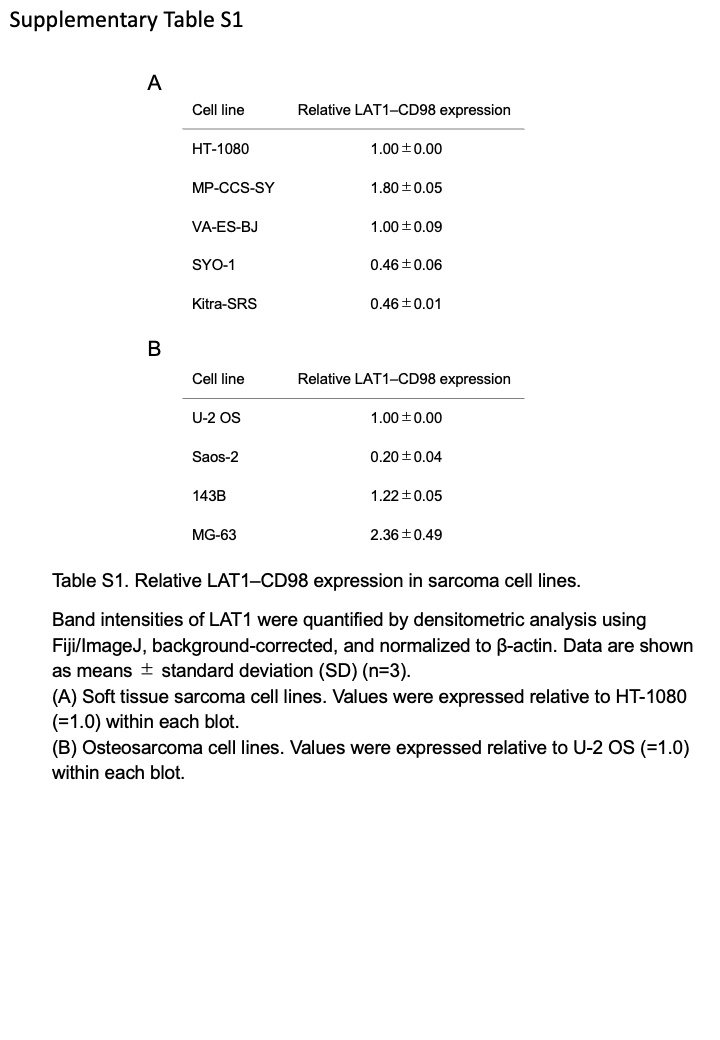

Supplement: Supplementary file 1 [file ijms-26-08599-s001.zip › Supplementary Table S1.tiff]

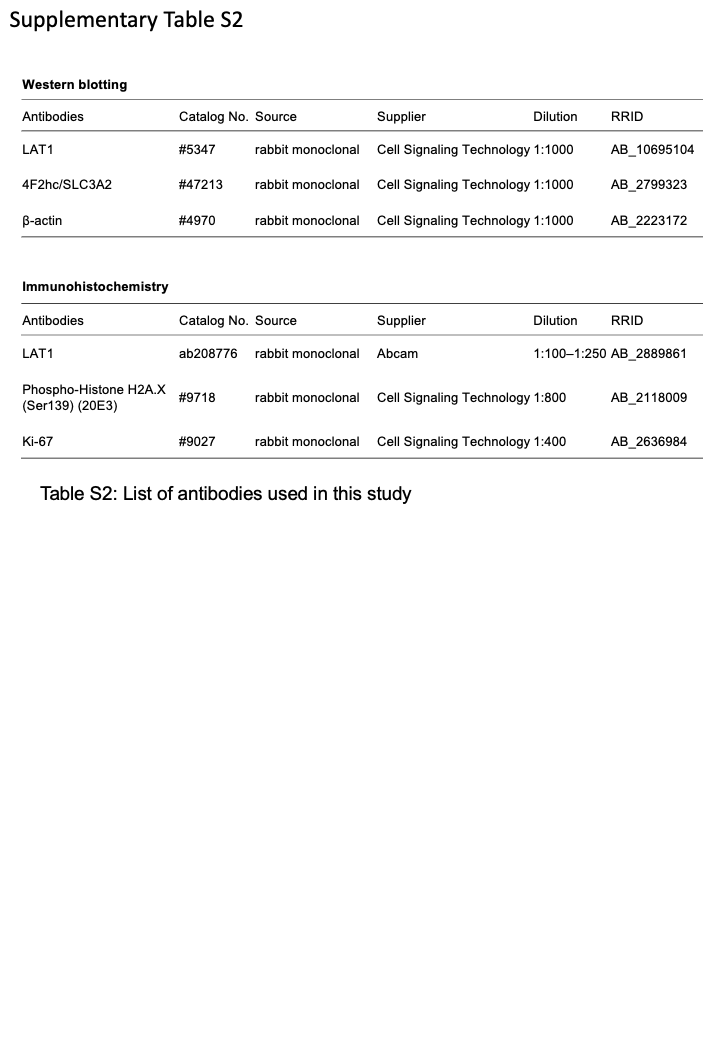

Supplement: Supplementary file 1 [file ijms-26-08599-s001.zip › Supplementary Table S2.tiff]
